# Immunohistochemical Expression of Survivin and Its Relationship with Cell Apoptosis and Proliferation in Ameloblastomas

**DOI:** 10.1155/2015/301781

**Published:** 2015-03-18

**Authors:** Rogelio González-González, Nelly Molina-Frechero, Pablo Damian-Matsumura, Sirced Salazar-Rodriguez, Ronell Bologna-Molina

**Affiliations:** ^1^Research Department, School of Dentistry, Universidad Juárez del Estado de Durango (UJED), 34000 Durango, Mexico; ^2^Doctorado en Ciencias Biológicas y de la Salud, Universidad Autónoma Metropolitana, 04960 Mexico City, Mexico; ^3^Health Care Department, Universidad Autónoma Metropolitana, Xochimilco, 04960 Mexico City, Mexico; ^4^Department of Biology of Reproduction, Universidad Autónoma Metropolitana, Iztapalapa, 09340 Mexico City, Mexico; ^5^Society for Fight Against Cancer, Portoviejo, 130105 Manabi, Ecuador; ^6^National Institute of Oncology and Radiobiology, 10400 La Habana, Cuba; ^7^School of Dentistry, Universidad de la República (UDELAR), 19200 Montevideo, Uruguay

## Abstract

Ameloblastoma behavior is related to the potential of tumor cells to inhibit apoptosis and to initiate a proliferative phase. This study was performed to compare the immunoexpression of Survivin with Bcl-2, Bax, and Ki-67 and to associate them with the histopathological type of each variant of ameloblastoma. *Material and Methods*. Using the World Health Organization (WHO) criteria for ameloblastoma, 110 cases were selected. The cases were classified as solid/multicystic and unicystic ameloblastomas. Cellular counts of cytoplasmic immunoexpression were assessed for cytoplasmic Survivin, Bcl-2, and Bax, while the nuclear immunoexpression of Survivin and Ki-67 was assessed using label index. *Results*. Cytoplasmic Survivin and Bcl-2 showed higher percentages of immunoexpression in solid multicystic ameloblastomas compared to unicystic ameloblastomas (*P* < 0.05). Bax, Ki-67, and nuclear Survivin were expressed in higher percentages in unicystic ameloblastomas. *Conclusions*. Cytoplasmic Survivin and Bcl-2 immunoexpression levels were elevated in relation to Bax immunoexpression, suggesting aggressive ameloblastoma behavior, while Ki-67 and nuclear Survivin immunoexpression may be associated with the type of tumor morphology that influences cellular counts or with the greater capacity for cellular proliferation and tumor growth.

## 1. Introduction

Ameloblastoma (AM) is considered one of the most frequent odontogenic tumors of epithelial origin and is a benign neoplasm with an aggressive behavior [1, 2].

In 2005, the World Health Organization (WHO) classified AMs as solid/multicystic (SMA), unicystic (UA), peripheral, or desmoplastic according to their clinical and histopathological characteristics [3]. SMA is an aggressive, slow growing and locally invasive neoplasm characterized by two main histopathological growth patterns, the follicular and the plexiform, and by cellular variations that do not impact the clinical behavior [3]. UA is classified according to cellular growth as intraluminal UA (IUA) and luminal UA (LUA), where the cystic wall remains uninvaded and the clinical behavior unaffected, or as mural UA (MUA), where the cells invade the cystic wall and, depending on the depth of invasion, affect the clinical behavior [3]. Apoptosis is a physiological process that maintains tissues during homeostasis [4]. During tumorigenesis, tumor cells may invade the proapoptotic processes by overregulating the antiapoptotic proteins [4]. Apoptosis inhibition is regulated by a group of molecules called the inhibitor of apoptosis proteins (IAPs), a group that includes Survivin [5–7]. Survivin is expressed during embryo-fetal development [8], is rarely expressed in adult tissue, and may be overregulated in proliferative processes [6–9]. Survivin is present in two subcellular compartments (the cytoplasm and the nucleus), plays antiapoptotic and promitotic roles, and takes part in Bax inhibition. Furthermore, this protein not only inhibits apoptosis but also promotes cell proliferation in malignant tumors [6, 10, 11].

The Bcl-2 protein family includes Bcl-2 and Bax, which play important roles in the regulation of the apoptosis process.* Bcl-2* is a protooncogene that codes for an internal mitochondrial protein, Bcl-2, an inhibitor of apoptosis [7, 8]. Bax is a proapoptotic protein that induces mitochondrial outer membrane permeabilization through translocation or insertion; causing membrane oligomerization, pore formation, and the release of mitochondrial proteins from the intermembrane space (i.e., cytochrome C, Smac/Diablo, and Omi/Htra2) [5, 12, 13].

The Ki-67 nuclear antigen is a widely used cell proliferation marker [14, 15] and a nuclear protein detected in all cell division phases except for the G0 phase [16, 17]. Ki-67 is frequently used to determine the growth fraction in different tumors [16–18], which is why a low growth fraction is associated with a favorable prognosis in several neoplasms.

The purpose of this study was to identify and to correlate cytoplasmic and nuclear Survivin immunoexpression with Bcl-2, Bax and Ki-67 immunoexpression in the SMA and UA variants.

## 2. Materials and Methods

### 2.1. Sample Tissues

The study group consisted of 110 cases of ameloblastoma from the Oral and Maxillofacial Pathology Service of public and private hospitals in Mexico, the Oral Pathology Service of the School of Dentistry of Juarez University of the State of Durango, the University of the Republic of Uruguay, the Pathology Services of the National Institute of Oncology and Radiobiology and Calixto Garcia Hospital in Havana, Cuba. All cases were reevaluated by two pathologists with experience in odontogenic tumors and following the 2005 WHO classification [3]. When two or more histological subtypes of AM were present, the most aggressive or predominant subtype was considered. Sections presenting severe inflammation in the connective tissue were excluded.

The study was evaluated and accepted by the Ethics Committee of the Faculty of Dentistry of Juarez University of the State Durango (Folio CE-FO-UJED-01-13).

### 2.2. Immunohistochemistry

Sections (3 *μ*m thick) were cut and placed in poly-L-lysine-coated slides. The sections were deparaffinized in a 60°C oven for 30 minutes and placed in xylol for 5 minutes. The sections were rehydrated in decreasing alcohol concentrations (absolute, 90, 70, and 50%) and washed in distilled water. To unmask the epitopes, the antigen recovery was performed with 10 mM sodium citrate solution with either high or low pH, depending on the characteristics of each antibody. This recovery was performed in a microwave pressure cooker with a maximum power of 750 W in two cycles of 5 minutes. The samples were then cooled to room temperature and washed with distilled water. The endogenous peroxidases were blocked with 0.9% hydrogen peroxide, and the samples were again washed with distilled water and phosphate-buffered saline solution (PBS, pH 7.4). The primary antibodies were incubated for 30 minutes for Survivin (Clone 12C4, 1 : 100, Dako Corp, Carpinteria, CA, USA), Bcl-2 (Clone 124, 1 : 50, Dako Corp, Carpinteria, CA, USA), Bax (Polyclonal, 1 : 150, Dako Corp, Carpinteria, CA, USA) and Ki-67 (Clone MIB-1, 1 : 50, Dako Corp, Carpinteria, CA, USA). Subsequently, the sections were incubated with the biotinylated anti-mouse/anti-rabbit secondary antibody and streptavidin/peroxidase complex (LSA-B + labeled streptavidin-biotin, Dako Corp., Carpinteria, CA, USA) for 30 minutes each. The reaction products were displayed with 3,3′-diaminobenzidine-H_2_O_2_ substrate (Dako Corp, Carpinteria, CA, USA). Fragments of tonsillar tissue were used as positive controls, and incubation with the primary antibodies was omitted for the negative controls.

Survivin, Bcl-2, and Bax proteins express immunohistochemically on the cytoplasm and the cytoplasmic membrane; therefore, the percentages of positive tumor cells were evaluated on all slides. The percentage of positive Survivin, Bcl-2, and Bax immunoexpression was calculated using a 10x optical microscope objective corresponding to an area of 5.3 mm^2^. The photomicrographs were taken with a digital camera (Olympus C-7070) from five random areas with abundant neoplastic ameloblastoma cells. The results were gathered according to the percentage of positive cells in each case.

The Ki-67 assessment was performed in selected neoplastic cell-rich areas on the SMA cases and throughout the cystic lines and mural follicles of the UA variants. The nuclear Survivin immunoexpression was evaluated in neoplastic SMA and UA cells where nuclear and nuclear/cytoplasmic immunoexpression was observed. In both cases (Ki-67 and nuclear and nuclear/cytoplasmic Survivin immunoexpression), the cell counts were performed as follows: microphotography with a 40x objective (five fields per case) was taken, the images were transferred to an image processor to aid in the counting (ImageJ 1.47d. Rasband W. ImageJ, National Institutes of Health, USA, http://imagej.nih.gov/ij/), and rack counting was performed using ImageJ and then manually using the cell counter method to obtain the cellular proliferation index (number of positive tumor cells/total number of tumor cells, expressed as a percentage). This technique was used in all cases.

### 2.3. Statistical Analysis

Statistical analysis was performed with Pearson's *χ* ^2^ method, using the expected values. The Kruskal-Wallis test was applied to detect the differences between ameloblastomas and Survivin, Bcl-2, Bax, and Ki-67 immunoexpression. Results with *P* ≤ 0.05 were considered significant. The results were tabulated and the data analyzed with SPSS 20.0 statistical software (SPSS Professional Statistics, SPSS Inc., Chicago, IL, USA).

## 3. Results

A kappa value of 0.96 between the two calibrated pathologists was first obtained by histologically classifying the AMs as SMA and UA following the WHO criteria; subsequently, the cases without agreement were reevaluated until total conformity was obtained. Of a total of 113 cases, three were excluded, as the evaluated tissue did not correspond with any variant of ameloblastoma. The remaining total study sample consisted of 110 ameloblastomas, of which 38 were SMA: six were diagnosed as follicular cases (FSA), ten as acanthomatous ameloblastomas (ASA), and 22 as plexiform ameloblastomas (PSA). Of the UA (*n* = 72), eight cases were luminal unicystic (LUA), 24 were mural unicystic (MUA), and 40 were intraluminal unicystic ameloblastoma (IUA).

**Figure 1:**
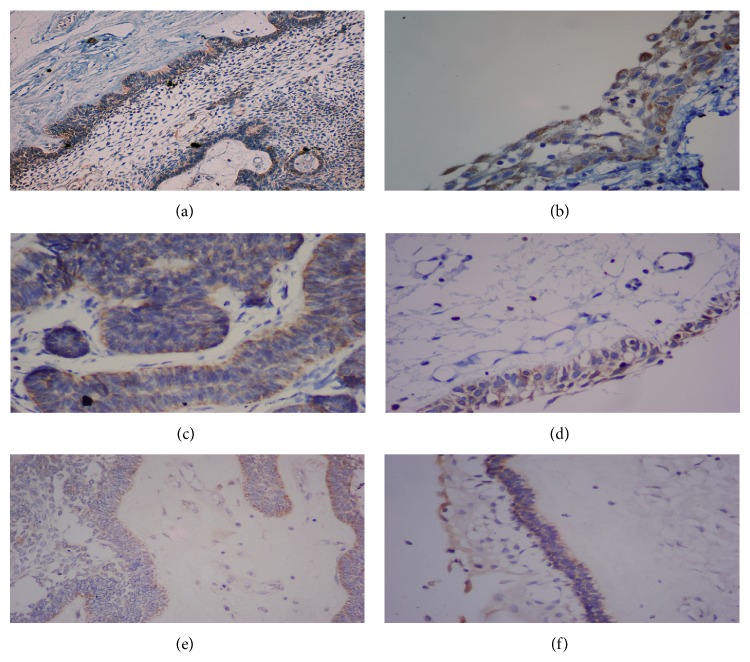
Differences in the cytoplasmic immunoexpression of Survivin, Bcl-2, and Bax in ameloblastomas. Differences in the cytoplasmic immunoexpression of Survivin in (a) solid/multicystic follicular ameloblastoma (100x) and (b) luminal unicystic ameloblastoma (400x). Differences in Bcl-2 immunoexpression in (c) solid/multicystic follicular ameloblastoma (400x) and (d) luminal unicystic ameloblastoma (400x). Differences in Bax immunoexpression in (e) solid/multicystic plexiform ameloblastoma (100x) and (f) luminal unicystic ameloblastoma (100x).

The positivity index for cytoplasmic Survivin was higher in the SMA than the UA samples (*P* < 0.05). Similar to cytoplasmic Survivin, the Bcl-2 positivity index was higher in the SMA samples (*P* < 0.05* versus* UA), while the UA cases showed a higher Bax positivity index (*P* < 0.05* versus* MUA) (Figure 1).

**Figure 2:**
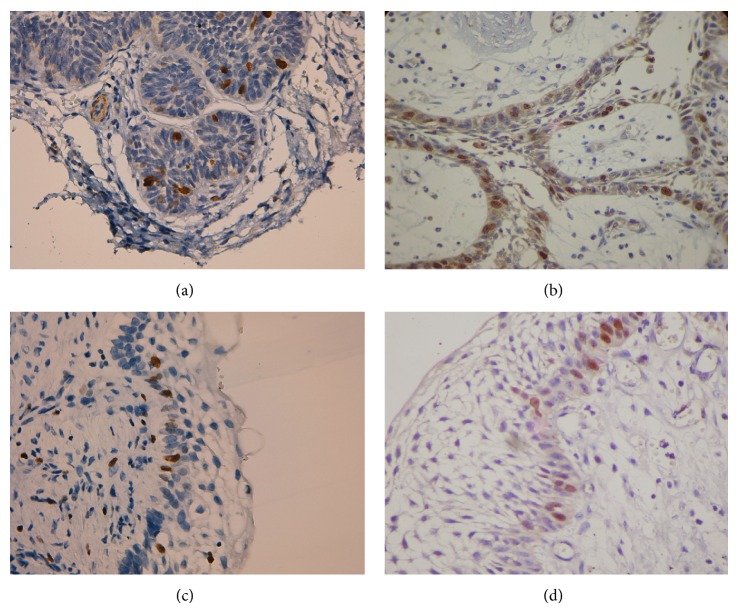
Ki-67 and nuclear Survivin immunoexpression. (a) Ki-67 immunoexpression in solid/multicystic follicular ameloblastoma (400x). (b) Nuclear Survivin immunoexpression in solid/multicystic plexiform ameloblastoma (400x). (c) Ki-67 immunoexpression in intraluminal unicystic ameloblastoma (400x). (d) Nuclear Survivin immunoexpression in intraluminal unicystic ameloblastoma (400x).

Table 1 shows the positivity index of the cellular apoptosis regulatory proteins among the AM histological subtypes. The UA cases had a higher cellular proliferation index for Ki-67 and greater nuclear Survivin immunoexpression than the SMA cases (*P* < 0.05) (Figure 2).  Table 2 shows the cellular proliferation index of Ki-67 and the nuclear immunoexpression of Survivin among the histological AM subtypes.

## 4. Discussion

Interestingly, UA was the predominant type of AM in our series, which is consistent with previous studies in Latin America [19, 20], while SMA are the most frequent AM types in North America and Asia [21]. Therefore, it is possible that this variant is predominant in some Latin American countries due to geographical, ethnic, and social differences; however, these results might be associated with a lack of uniform diagnostic criteria or with the inclusion of incision biopsies and surgical resections that could explain the variation in the frequency of the AM subtypes.

Several studies have identified molecular alterations associated with AM development and progression [22], including those associated with proteins involved in the apoptotic and cellular proliferation pathways [23, 24].

Survivin is a protein from the IAP family that regulates cellular death, suppresses cellular apoptosis, and regulates cellular division [25]. High Survivin immunoexpression is present in several malignant neoplasms [26–29].

In our study, SMA exhibited higher cytoplasmic Survivin immunoexpression in the ameloblastic tumor cells than in UA. Furthermore, Survivin immunoexpression was higher compared with Bax immunoexpression in SMA, while in UA, Bax immunoexpression was higher than Survivin immunoexpression.

The Bcl-2 and Survivin proteins are involved in the inhibition of apoptosis processes and possibly play roles in cellular proliferation, tumor progression, aggressive clinical behavior, and oncogenesis of the odontogenic epithelium [1, 10]. Similar to cytoplasmic Survivin, Bcl-2 inhibits the proapoptotic functions of Bax and promotes cellular proliferation, which is ultimately reflected in the biological tumor behavior of each type of AM [23, 24]. The Bcl-2 protein has been identified in B-cell non-Hodgkin follicular lymphomas and is an important regulator of programmed cell death. The positive immunoreaction of the Bcl-2 product is observed in long-lived cellular populations or in those with a proliferative high capacity [23, 24]. In this study, a higher percentage of Bcl-2 immunoexpression was observed in ameloblast-like tumor cells, confirming the high Bcl-2 protein immunoexpression of either most or a considerable number of ameloblastic tumor cells; our results are in agreement with published data from other authors and could be considered as a type of inhibition of the mechanisms of cellular apoptosis induction [24, 30, 31].

According to Mitsuyasu et al. [31], the Bcl-2 protein significantly inhibits AM tumor cell apoptosis, and our results could support this suggestion. The high percentage of Bcl-2 immunoexpression in AM cells, especially in basal cells with inverse polarization, possibly affects the behavior of the AMs. In this study, SMA had a higher percentage of Bcl-2 immunoexpression than did UA.

However, Bax is a promoter of apoptosis, is similar to Bcl-2 at the amino acid level and is capable of forming homodimers and heterodimers with Bcl-2. The relationship between Bcl-2 and Bax determines the range of survival or cellular death. When Bax predominates Bcl-2 activity, it becomes repressed, and the apoptotic pathway of Bax is activated [30, 32].

Bax immunoexpression was lower in SMA than in UA. This result suggests that SMA tumor cells inhibit apoptosis due to the difference in Bcl-2 and Bax immunoexpression levels. The relationship between these two proteins would justify the more aggressive biological behavior of SMA. Sandra et al. [24] and Soluk Tekkeşin et al. [30] reported that over-immunoexpression of anti-apoptotic proteins in the odontogenic epithelium is linked to cellular proliferation, AM cell differentiation and apoptosis inhibition, influencing on the clinical behavior of the AM.

Survivin can be detected in the nuclei of tumor cells, which may generally indicate a poor prognosis in several malignant tumors [26, 28]. Furthermore, it is associated with metastasis to lymph nodes in oral and oropharyngeal cancers [28], suggesting an involvement of nuclear and cytoplasmic Survivin immunoexpression in cellular proliferation, poor cellular differentiation, and metastasis [26].

To the best of our knowledge, this is the first evidence of localization of Survivin protein in the nucleus of ameloblastic cells. In this study, nuclear Survivin immunoexpression was slightly exclusive in UA and only expressed in the plexiform variant of SMA. Stasikowska-Kanicka et al. [6] suggested that nuclear Survivin immunoexpression promotes cell proliferation, while cytoplasmic immunoexpression is involved in the mechanisms of apoptosis regulation. The nuclear Survivin immunoexpression was the most novel result that was found in this study. However, the percentage of nuclear positivity was very low, so more studies focusing on nuclear Survivin levels are needed to clarify whether nuclear Survivin could really be involved in promoting cell proliferation.

The Ki-67 protein is present in proliferative cells and, as with nuclear Survivin, an increased proliferation index indicated poor prognosis. The results involving Ki-67 in this study are in agreement with Meer et al. [33], Rosenstein et al. [34], and Bologna-Molina et al. [20], suggesting that the immunoexpression index of Ki-67 in UA could be associated with bone destruction and aggressive behavior; however, the differences in tumor morphology may be the reason for this finding, as SMAs form large follicles or plexiform nests, while UAs present only unicystic areas with thin epithelia, thus including great numbers of basal and suprabasal tumor cells with a subsequent increase in the cellular proliferation index. In other words, such a difference in the cellular count could be associated with a smaller number of stellate reticulum-like cells; thus, most cells correspond to the basal and suprabasal layers that have a greater tendency to proliferate and have higher levels of Ki-67 nuclear immunoexpression [20].

The correlations between Survivin and Bax/Bcl2/Ki-67 have already been done in many types of tumors. Although the immunoexpression of the proteins included in this study has already been studied in several malignancies, there exists little information in ameloblastic and odontogenic tumors. Our work has the advantage of including one of the biggest ameloblastoma series reported.

However, our study has the limitations of using only a single experimental approach and the lack of comparison with a malignant odontogenic tumor. It is necessary to perform, a second confirmatory technique for nuclear Survivin and functional studies to analyze the presence and biologic role of nuclear Survivin protein in tumorigenesis and in the biologic behavior of ameloblastomas and other odontogenic lesions.

## 5. Conclusion

In conclusion, our data show that cytoplasmic expression of Survivin and the expression of Bcl-2 and Bax are relate with behavior of ameloblastomas and possibly the higher expression for Ki-67 and nuclear Survivin in UA are associate with the epithelial morphology. Future studies in the mechanisms associated with apoptosis and with cell proliferation process in ameloblastic tumors, including malignant tumors, such as ameloblastic carcinomas, are required to help to clarify the association of these mechanisms in the tumoral biological behavior.

## Figures and Tables

**Table 1:** Average expression percentages of apoptosis regulatory proteins according to the ameloblastic histological types and subtypes.

Histological type	Number of cases	Mean ± SD		
		%Cytoplasmic Survivin	%Bcl-2	%Bax
Solid/multicystic	38	45 ± 31.6	46 ± 30^a^	40.2 ± 25.8
FSA	6	43.3 ± 34.4	58.3 ± 29.2	46.6 ± 30.1
ASA	10	43 ± 27.5	58 ± 30.4	46 ± 22.7
PSA	22	46.3 ± 33.8	37.2 ± 28.3	35.9 ± 26.3
Unicystic	72	39.5 ± 29.7	40.2 ± 27.3^b^	43.1 ± 25.4
LUA	8	27.5 ± 30.1	33.7 ± 19.2	37.5 ± 31.1
MUA	24	44.6 ± 34.6	43.3 ± 26.6	48.3 ± 24.1
IUA	40	39 ± 29.6	36.7 ± 28.3	41.2 ± 25.3^c^

FSA: follicular solid/multicystic ameloblastoma, ASA: acanthomatous solid/multicystic ameloblastoma, PSA: plexiform solid/multicystic ameloblastoma, LUA: luminal unicystic ameloblastoma, MUA: Mural unicystic ameloblastoma, IUA: intraluminal unicystic ameloblastoma. Kruskal-Wallis test; the *P* values in bold indicate statistical significance. (*P* < 0.05). Solid/multicystic ameloblastoma: ^a^%cytoplasmic Survivin, vs. %Bcl-2, *P* = 0.007, Unicystic ameloblastoma: ^b^Bax, vs. Bcl-2, *P* < 0.05, Intraluminal ameloblastoma: ^c^%cytoplasmic Survivin vs. %Bax, *P* < 0.05.

**Table 2:** Average percentages of Ki-67 and nuclear Survivin expression according to ameloblastic histological types and subtypes.

Histological type	Number of cases	Mean ± SD	
		%Ki-67	%Nuclear Survivin
Solid/multicystic	38	12.8 ± 10.4	0.45 ± 1.3
FSA	6	11.1 ± 6	—
ASA	10	9.9 ± 7.4	—
PSA	22	14.6 ± 14.3	0.77 ± 1.7
Unicystic	72	16.4 ± 14.3	0.72 ± 1.5
LUA	8	24.1 ± 17.8	0.5 ± 1.4
MUA	24	17.1 ± 17.8	0.67 ± 1.7
IUA	40	14.5 ± 10.9	0.8 ± 1.6

FSA: follicular solid/multicystic ameloblastoma, ASA: acanthomatous solid/multicystic ameloblastoma, PSA: plexiform solid/multicystic ameloblastoma, LUA: luminal unicystic ameloblastoma, MUA: mural unicystic ameloblastoma, IUA: intraluminal unicystic ameloblastoma. Kruskal-Wallis test. No statistical significance was observed between Ki-67 and Nuclear Survivin, in subtypes of ameloblastomas.
